# Obstacles and Recommendations for Clinical Translation of Nanoparticle System-Based Targeted Alpha-Particle Therapy

**DOI:** 10.3390/ma14174784

**Published:** 2021-08-24

**Authors:** Janke Kleynhans, Mike Sathekge, Thomas Ebenhan

**Affiliations:** 1 Division of Nuclear Medicine, Tygerberg Hospital, Stellenbosch University, Cape Town 8000, South Africa; jk1@sun.ac.za; 2 Department of Nuclear Medicine, University of Pretoria & Steve Biko Academic Hospital, Pretoria 0001, South Africa; mike.sathekge@up.ac.za; 3 Nuclear Medicine Research Infrastructure NPC, Pretoria 0001, South Africa; 4 Department of Nuclear Medicine, University of Pretoria, Pretoria 0001, South Africa

**Keywords:** nano-generators, nanoparticles, liposomes, recoil energy, nanotechnology regulatory challenges, oncology, physical half-life, radiochemistry, theranostics, nanotoxicology

## Abstract

The rationale for application of nanotechnology in targeted alpha therapy (TAT) is sound. However, the translational strategy requires attention. Formulation of TAT in nanoparticulate drug delivery systems has the potential to resolve many of the issues currently experienced. As α-particle emitters are more cytotoxic compared to beta-minus-emitting agents, the results of poor biodistribution are more dangerous. Formulation in nanotechnology is also suggested to be the ideal solution for containing the recoil daughters emitted by actinium-225, radium-223, and thorium-227. Nanoparticle-based TAT is likely to increase stability, enhance radiation dosimetry profiles, and increase therapeutic efficacy. Unfortunately, nanoparticles have their own unique barriers towards clinical translation. A major obstacle is accumulation in critical organs such as the spleen, liver, and lungs. Furthermore, inflammation, necrosis, reactive oxidative species, and apoptosis are key mechanisms through which nanoparticle-mediated toxicity takes place. It is important at this stage of the technology’s readiness level that focus is shifted to clinical translation. The relative scarcity of α-particle emitters also contributes to slow-moving research in the field of TAT nanotechnology. This review describes approaches and solutions which may overcome obstacles impeding nanoparticle-based TAT and enhance clinical translation. In addition, an in-depth discussion of relevant issues and a view on technical and regulatory barriers are presented.

## 1. Introduction

Whilst a considerable number of published preclinical evaluations demonstrate the positive influence of the formulation of α-particle emitters in connection with nanoparticles, their successful clinical translation is lacking. The benefits of nanoparticles for the delivery of radionuclides (therapeutic and diagnostic) are well-substantiated and reviewed [1,2,3]. However, a critical evaluation of the maturity level of those techniques is required. The focus of research involving targeted alpha therapy (TAT) is related to treatment of malignancy by achieving maximum therapeutic efficacy with low damage to non-target tissue [4]. Most of the early research on therapeutic radiopharmaceuticals was aimed at β-minus particle-emitting radionuclides, and indeed, some was successfully translated into the clinic. The most relevant example is [^177^Lu]Lu-DOTA-TATE (DOTA-Tyr^3^-Octreotate) therapy for neuroendocrine malignancy expressing somatostatin receptors [5]. The physical limitation of β-particle-emitting radiopharmaceuticals is their long energy emission range, that can often lead to damage or death in healthy cells within the vicinity of the targeted pathologic tissue. These types of therapies are consequently less appropriate for smaller (micro-metastatic) cancerous foci. TAT on the other hand deposits energy within a few cell diameters, theoretically leading to a less toxic therapy. TAT also provides a very high relative biological effectiveness with an approximate 500 times more cytotoxic potential compared to β-particle emitters [1,4].

**Figure 1:**
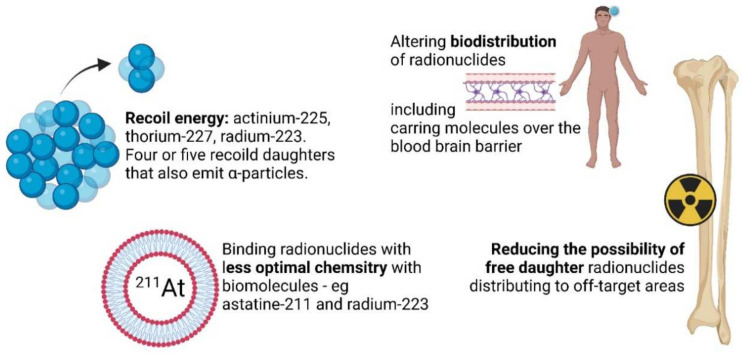
Shortcomings in TAT and addressing strategies using nanotechnology.

The main α-particle-emitting radionuclides used for TAT are radium-223, radium-224, astatine-211, actinium-225, lead-212, thorium-227, thorium-227, bismuth-212, and bismuth-213. Their main nuclear properties have been summarized in several reviews by Majokwska-Pilip et al. [1] and Farzin et al. [2]. Nanoparticles are used in TAT, predominantly: (i) to reduce the release of radioactive daughters from targeting vectors, (ii) to circumvent the lack of appropriate ligands for the effective binding of α-particle emitters to targeting ligands, (iii) to reduce the distribution to off-target areas, and (iv) to alter biodistribution of the radionuclides (Figure 1). There are also prospects for TAT nanoparticulate systems to provide new therapeutic approaches for nano-brachytherapy.

This review describes strategies which may be employed to diminish impediments to nanoparticle-based TAT and thereby enhance clinical translation. In addition, an in-depth discussion of relevant issues and a view on technical and regulatory barriers are presented. Although certain α-generators are referred to as in vivo atomic nanogenerators [4] in their own right (actinium-225, thorium-227, and radium-223), this review focuses on the application of nanotechnology on α-particle emitters and not in vivo atomic nanogenerators.

## 2. Current Challenges Using Targeted Alpha Therapy

### 2.1. Production Aspect and Limitations of Physical Half-Life

The first α-particle emitter approved was radium-223-chloride in 2013, and the application of these agents in oncology is therefore relatively new. It must be kept in mind that radium-223 targets bone metastases in its natural state based on the inherent molecular properties of this radionuclide and does not require manipulation to target osteoblastic bone lesions. Other α-particle emitter treatments that have gained traction clinically are [^225^Ac]Ac-PSMA-617 and [^225^Ac]Ac-DOTA-TATE therapies [6,7,8,9]. Currently, improved understanding of α-particle emitter therapy, discovery of new tumor targets, understanding of molecular status of malignancy, and the availability of new ligands has generated considerable interest from the nuclear medicine and oncology communities. The intrinsic physical half-life of α-particle emitters limits the candidate radionuclides for application in nuclear medicine. From more than 100 α-particle emitters known to mankind, the majority have inappropriate half-lives or energy profiles. Furthermore, most of the useful α-particle emitters have uneconomical production methods. The α-particle emitters that are currently highlighted for TAT are radium-223 (t½ = 11.4 days), astatine-211 (t½ = 7.2 h), actinium-225 (t½ = 10 days), thorium-227 (t½ = 18.7 days), bismuth-212 (t½ = 60.6 min), bismuth-213 (t½ = 45.6 min), lead-212 (t½ = 10.6 h), and terbium-149 (t½ = 4.1 h). Of these, astatine-211, actinium-225, thorium-227, radium-224, and lead-212 are of interest in nanoparticle applications because their half-lives are more appropriate and compatible with the known pharmacokinetics of nanoparticles (refer to Table 1). Another well-known challenge that affects the TAT technological applicability is the actual availability of α-particle emitters. Currently, production capacity is inadequate for the clinical demand. For example, the amount of actinium-225 produced per year can only provide about 5000 doses [10]. However, around 100,000 patients per year in Europe alone could benefit from TAT, clinically accessible subject to the availability of actinium-225. These employ small-molecule radiopharmaceuticals such as actinium-225 for prostate therapies and actinium-225 for somatostatin receptor 2 targeting therapy. Another prime example of poor technological preparedness regarding radionuclide production is astatine-211, where the lack of radioisotope availability, expensive infrastructure, and difficult isolation techniques result in substantial obstruction of its clinical use beyond small-scale clinical trials or proof-of-principle studies. Solutions to alleviate the problem have been described in some detail in several recent literature reviews [10,11,12,13], which underline the demand for progress in this area of research and engineering. Considering that these radionuclides require purification after production and that incorporation into nanoparticles is most often a more arduous process than other radiolabeling strategies, only α-particle emitters with half-life longer than or equal to astatine-211 (t½ = 7.2 h) are considered suitable for inclusion into nanoparticles [14,15]. However, this is debatable as several nanoparticle systems incorporate diagnostic radionuclides with much shorter physical half-lives (e.g., gallium-68 (t½ = 68 min)) [16,17]. Nonetheless, the collective literature on TAT nanotechnology reports that most of the research involves α-particle emitters with physical half-lives in the order of days, and it is advisable to focus on these radionuclides for more flexibility during the production process. Generally, a longer physical half-life reduces waste due to decay during radiochemical processing and distribution of the radiopharmaceutical [18]. It is also extremely important that the physical half-life of the selected α-particle emitter ideally matches the pharmacokinetic properties of the nanotechnology chosen for delivering it to its target.

### 2.2. Chemistry Constraints

The use of nanoparticles is a suggested strategy to address the disadvantages of α-particle emitters that possess ill-fitted chemistry for their incorporation or complexation into useful targeting vectors. For example, astatine-211 has unique properties by way of chemically resembling iodine and also behaves as a metalloid due to its position on the periodic table. Most radiopharmaceuticals incorporating this radionuclide are hampered by in vivo stability, mainly due to the weakness of the astatine–biomolecule bond [42]. The radiochemistry of radium-223 also suffers from its suboptimal chemical characteristics. Since radium is part of the alkali earth group on the periodic table, this element (Ra^2+^) forms very weak complexes. Incorporation of radium-223 into biomolecules has, to date, been unsuccessful. To establish applications of the unformulated radionuclide radium-223, other than bone metastasis therapy, nanoparticle-based chemistry seems to provide better radiolabeling characteristics [43].

One of the challenges of TAT that available nanotechnology does ameliorate is the concern about organ toxicity caused by inappropriate leakage of radionuclides from the bioconjugate. When certain TAT radiopharmaceuticals are administered, α-particle emitters can become uncoupled (or trans-chelated) from their metal-chelating moiety, i.e., they become free to distribute to off-target areas and may cause perpetual toxicity. For example, it is known that high levels of unbound actinium-225 can distribute to the liver and may lead to organ damage. Free astatine-211 is distributed to the thyroid and unbound bismuth-221 is known to cause renal complications. Lead-212 and radium-223 both distribute within the gastrointestinal system, where abdominal pain, nausea, diarrhea, vomiting, and peripheral oedema are caused [18].

### 2.3. Pharmacokinetic Behavior

Concerns about potential toxicity are also related to the release of daughter radionuclides. An example is the daughter radionuclide bismuth-213 (mother: actinium-225), that accumulates in the renal-cortex. Furthermore, bone marrow suppression can be caused from thorium-227, because of its mother radionuclide radium-223 that accumulates on the bone surface [4,7].

**Figure 2:**
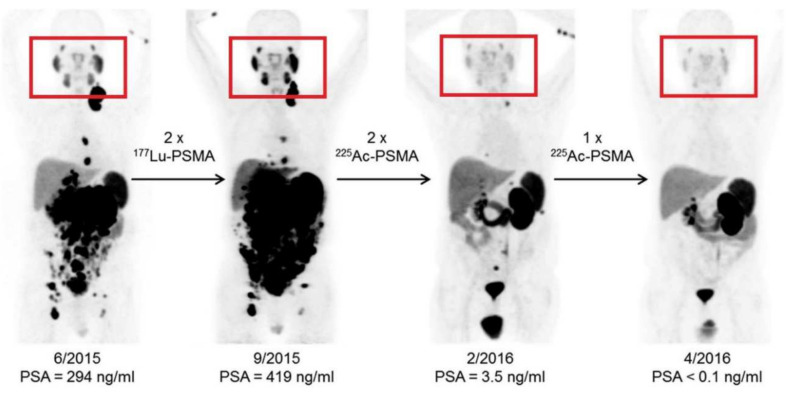
The effect of [^225^Ac]Ac-PSMA-617 treatment on salivary glands compared to [^177^Lu]Lu-PSMA-617 treatment (this research was originally published in JNM. Kratochwil et al. [46], ^225^Ac-PSMA-617 for PSMA targeted α-radiation therapy of metastatic castration-resistant prostate cancer. *J. Nucl. Med.* **2016**, *57*(12), 1941–1944©SNMMI).

It is well-understood that significant differences in biodistribution of the radionuclide can be brought about by the formulation of the TAT in nanotechnology. Currently, a lot of research is geared towards solving the unwanted side-effects of TAT. A most notable example is severe xerostomia as well as lacramal gland toxicity associated with [^225^Ac]Ac-PSMA-617-TAT (Figure 2). Current strategies to prevent [^225^Ac]Ac-PSMA-617-TAT-toxicity are using larger molecules (PSMA-targeting antibodies) to deliver the TAT, using external cooling with icepacks, applying botulinum toxin or administering chemicals such as phosphonomethyl-pentanedioc acid (2-PMPA) or polyglutamate [8,32]. Many of these strategies have been applied, with various levels of success. Although many chemical alterations on PSMA ligands continue to be investigated, nanoparticles remain a feasible option. pH-sensitive liposomes are a well-studied system [44] and PSMA-targeting liposomes have also been developed [45] with a similar objective in mind.

## 3. Radiolabeled Nanoparticle-Based Systems Applicable to TAT

Nanotechnology is often referred to as technologies between 1 and 100 nm in scale, and nanomedicine applies these nanosized structures to improve therapeutic efficacy. Due to their size, these nanoparticles have unique physical, chemical, and biological properties [24].

**Figure 3:**
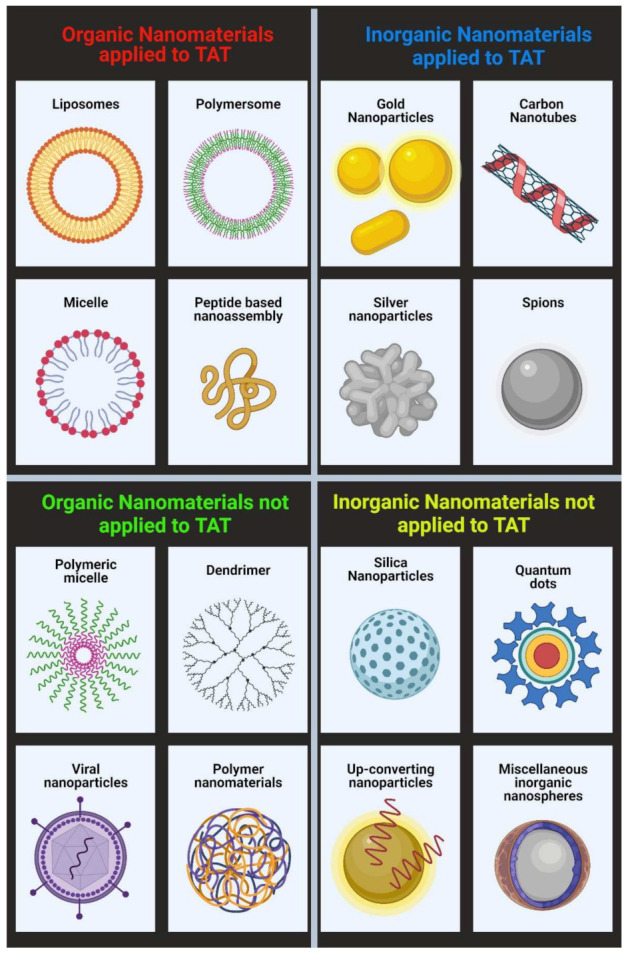
Different types of nanomaterials (non-exhaustive) including examples applied to TAT and examples yet to be applied to TAT (Figure created with BioRender.com (accessed on 12 April 2021)).

It is beyond the scope of this review to discuss all the different types of nanotechnology and their production methods. However, a relevant summary is provided in Figure 3. Radiopharmaceutical production and the types of nanoparticles of interest have been extensively reviewed by Farzin et al. [2].

There is significant interest in the incorporation of α-particle emitters into the various available technologies and in the progress of TAT and nano-radiopharmaceutical integration. There are a few major methods that can be used for nano-radiopharmaceutical production. Firstly, the radionuclide can be incorporated during the manufacturing of the nanocarrier (“inner incorporation”). This is normally more appropriate for radionuclides with longer physical half-lives as this method dictates that the radionuclide should be present during the whole of the nanoparticle manufacturing process. The second method of nanoparticle radiolabeling is to functionalize the surface of the nanoparticle in such a way that it can be radiolabeled after manufacturing by surface labeling (“surface coupling”). The radionuclide will consequently be included on the surface of the nanoparticle. This may be done by using a prosthetic group, chelator, or direct surface labeling. A third route is to incorporate the radionuclide after preparation by a passive loading system. The nanoparticle can for example be incubated with a concentration of radionuclide, which subsequently moves passively into the nanoparticle. A final labeling option is called “after-loading”, where the radionuclide is loaded into the nanoparticle by an active physicochemical interaction after manufacturing. This may be achieved by using a gradient across the nanoparticle surface with a chelator inside the nanoparticle which entraps the radionuclide. Ionophores offer another system that is used successfully. Alternatively, nanoparticles can be made from radioactive material or be irradiated after manufacturing, but to date, none of these methods have been applied to TAT [47]. These methods of nanoparticle radiolabeling are becoming better understood, as evident from the in-depth information reported in recent reviews [48,49,50].

The advantages of using nanoparticles for the delivery of therapeutic radionuclides such as α-particle emitters are numerous. Nanomaterials can be engineered to enhance local accumulation at tumor cells and lead to high within-tumor retention. Similarly, nanomaterials may incorporate multi-modalities, enabling other diagnostic radionuclides or other imaging modality agents to be incorporated within the TAT. This is especially important in the light of TAT radiation dosimetry difficulties. Nanotechnology may increase the internalization of TAT in tumor cells, especially in the case of low levels of receptor expression. Furthermore, additional delivery mechanisms (for instance, external magnetic field applications) can be integrated into nanotechnology applications to increase delivery of α-particle emitters. The technology to adjust the pharmacokinetics of nanoparticulate delivery systems is also well-developed [51].

The chosen radiolabeling conditions as well as nanotechnology applications must be matched with the radionuclide’s physical half-life. The pharmacokinetic profile of the nanomaterial vehicle is also a significant factor, and it must be matched with the selected radionuclide and targeting vector. For instance, radionuclides with long physical half-lives might be optimal for differential tumor accumulation and cellular internalization, but it should be ensured that they are not distributed to non-target sites [52]. As apparent from the current summary of preclinically evaluated TAT nanotechnology systems as presented in our review, these designed concepts have been shown to be valid. As pointed out by Sindhwani and Chan [47], a solid foundation is in place and strategies are needed to accelerate translation. Currently, most projects are led by engineers and chemists [48], with little input from the clinical team (Radiopharmacists, Nuclear Medicine Technologists, Biochemists, Radiobiologists, and Nuclear Medicine Physicians). Basic sciences tend to rationalize design from a technology perspective without enough focus on the behavior of these systems in human subjects and possible clinical applications [47]. Consequently, it is important that technology and clinical partnerships are considered simultaneously to result in optimal smart radio-nanotechnology to solve the problems associated with TAT.

The following basic prerequisites are suggested for the successful development of a nanoparticulate therapeutic agent, system, or strategy [53]: (i) a predictable drug incorporation and release (i.e., effective loading inside NP), (ii) an adequate formulation stability and shelf-life, (iii) sufficient biocompatibility and non-toxic character, (iv) desirable biodistribution and targeting (including sufficient residence time), (v) known functionality, (vi) knowledge of adverse effects of residual material—measuring persistence, (vii) internalization and drug release—complete biodegradation and elimination of NP, and (viii) validated quality control methods and robust manufacturing, including stringent characterization.

Additionally, specifically pertaining to TAT, it is suggested that the following physio-chemical objectives are achieved: (i) demonstration and ability of the successful entrapment of all daughter radionuclides, (ii) demonstration of stable chemical associations that can withstand in vivo conditions, and (iii) preferably accommodate a theranostic partnership to determine viability of the treatment strategy.

An approach that uses nanometer-sized zeolites [36,54] is worth mentioning. The Na A-type zeolite hereby provides high selectivity for Ra^2+^-ions, and radium-223 is well-retained [55]. When gold-based nanoparticles are evaluated for entrapping α-particle emitters, their size should be around 36 nm to retain all the daughter radionuclides (up to the third daughter) and conquer recoil energy. Lastly, it is now evident that liposomal nanoparticles do not possess enough structural integrity to counteract the recoil energy and retain any possible daughter radionuclides. For further details, refer to the review by Holzwarth et al. [55].

## 4. Pitfalls for Bench-to-Bedside Translation

### 4.1. Large-Scale Manufacturing

Nanotechnology is designed to improve the active pharmaceutical ingredient (or radionuclide) system’s biodistribution, stability, targeting, and retention, as well as to introduce theranostic approaches [52]. A challenge that hampers the development and large-scale production of efficient pharmaceutical nano-carriers is the lack of structure-controlled manufacturing methods that will allow the cost-effective and robust routine production of nano-pharmaceuticals [56]. Every nanotechnology sub-type has associated issues that may delay clinical application. For example, liposomes are hampered by a low encapsulation efficiency, leakage of water-soluble drugs in vivo (due to the presence of blood components), and a tendency to aggregate. Liposome manufacturing anomalies involve deviations in specifications as the result of process-upscaling, lack of Good Manufacturing Practice (GMP) procedures, and issues with batch-to-batch reproducibility [56]. To manufacture nanotechnology on GMP standards results in the need for upscaling that will ensure the same product (zeta-potential, size distribution, entrapment, etc.) as what was tested in preclinical and early clinical evaluations. Normal radiopharmaceutical tests such as endotoxin testing, filter integrity testing, and radiochemical purity determination may not be translatable to TAT nanoparticles due to the nature of the technology influencing the quality control (QC) tests. In such instances, equivalent tests may need to be found and validated to provide equally acceptable results. It is important to note that obtaining GMP and the QC and QA (quality assurance) of nanomaterials will be much more demanding than that of the classical low molecular weight radiopharmaceuticals, and there will be a higher economic and manpower burden on the facility to ensure that standards are met.

Similar issues are prevalent in other types of nanocapsule production, for example polymer micelles, polymersomes, dendrimers, and polymeric nanoparticles [57]. Inorganic-based nanoparticulate systems may contain component chemicals that are not soluble within biological matrices and persist in vivo [53], potentially producing a toxic accumulation of inorganic particles. Even when nanoparticles are designed effectively and evaluated extensively, the challenges may not end there. Any slight changes in raw materials or minor deviations from validated manufacturing procedures potentially cause variations in the characteristics of the final nanoparticle. Whilst these modifications are considered subtle, they can substantially affect nanoparticle biodistribution and biological properties in vivo [58]. The production of radiopharmaceuticals that include nanoparticles are therefore more risk-prone than other (more conventional) radiopharmaceutical products.

### 4.2. Size of Nano-Constructs

A well-validated size of nano-constructs is key as larger molecular vectors have been demonstrated to be taken up by the reticuloendothelial system and have slower and less optimal in vivo kinetics [29]. Nanoparticles, if not properly designed, suffer from nonspecific splenic and hepatic accumulation as they target the cell’s reticuloendothelial system. Crucially, the minimum size for particles must be greater than 10 nm to avoid first-pass renal filtration. The upper limit is considered at 150 nm, but other factors such as particle shape, surface characteristics, and presence of functional targeting moieties also alter their biodistribution [52,59]. During TAT, it is critical that splenic, hepatic, and pulmonary nanoparticle accumulation is minimized to prevent unacceptable toxicity profiles, which is a particular complication that remains to be resolved to date [1].

### 4.3. Regulatory Challenges

The regulatory challenges pertaining to nanomaterials for medical application is a topic that deserves a more in-depth analysis. The first regulatory issue to require resolution was the regulatory and scientific definition of nanomaterial, where The European Commission defines nanoparticles as either “natural, incidental, or manufactured materials” that comprise materials that (unbound or aggregated) “consist of 50% or more particles having a size between 1 and 100 nm” (according to number size distribution). Any structure with one or more external dimensions less than 1 nm should be considered a nanomaterial. Materials with a surface area >60 m^2^/cm^3^ also fall within this definition [60]. The next issue confronting research is the complexity of Intellectual Property (IP). Generally, IP is controlled by the described encapsulated cargo, the carrier technology, and the characteristics of the active ingredient-carrier system, together. To date, however, in practice, the three criteria seem to be insufficient to alleviate issues regarding the applications for patents [61]. It is important that the nanomaterial be characterized through all design stages, and it must be ensured that any interaction with biological systems, sample preparations, or extraction procedures does not modify the properties of the system or influence measurements [60]. As such, pharmaceutical development of nanoparticles must focus greatly on the control of the manufacturing process and the identification of critical parameters that influence the biological behavior and toxicology of these nanoparticles. It is imperative that quality control procedures are robust and validated. It is also essential that quality-by-design pharmaceutical approaches (such as those prescribed by the FDA’s cGMP principles) are adhered to for the systemic evaluation and control of nanomedicines [60,62]. It is of note that the first generation of pharmaceutical nanotechnology products passed regulatory approval by adhering to the generic standards applicable to all medicinal compounds. These regulations are no longer viewed as appropriate and current approval is mostly provided on a case-by-case basis [61]. On this note, biological nanomedicines are regulated under the framework of the European Medicines Agency (EMA), which is adequate for quality in non-clinical and clinical studies. The regulatory approach for non-biological complex drugs is substantially more complicated and is frequently analyzed on a case-by-case basis by the EMA, which has created an expert group on nanomedicine, combining members from both academia and the European regulatory network [60]. Further details regarding the regulatory aspects pertaining to nanotechnology for medicinal use have been reviewed by Soares et al. [60], Jones et al. [62], and Hua et al. [61], and the authors recommend that readers regularly follow developments in this area. It is also critical to note that even traditional radiopharmaceuticals not using nanotechnology entail additional regulatory complications (reviewed by Decristoforo et al. [63]).

### 4.4. Toxicity

When the prospects of nanotechnology for TAT are evaluated, it is important that none of the literature regarding pharmaceutical application regarding the use of nanoparticles is ignored. It is widely assumed that nanoparticles are used as carrier systems to reduce the toxicity and side-effects of the active ingredient. However, often, no additional investigations addressing the carrier system itself and how it may pose a new risk to the patient are conducted. Any potential toxicity depends to a large extent on the actual composition of the nanoparticle and also the surface characteristics of this new entity [53].

## 5. Toxicity and Tolerability of Nanoparticulate Systems

Nanotoxicology must be given significant consideration during the design process and TAT incorporation. Some nanomaterials are developed with unique surface properties that are not comparable to bulk material.

Although the bulk material might be non-toxic, a nanoparticle made from the same material exhibits a whole set of novel, sometimes toxic characteristics. The lack of a set of standard criteria to cover the toxicity evaluations of all nanoparticles is concerning. In all cases, it is important that there is discrimination between drug toxicity (radiopharmaceutical), the nanoparticle toxicity, as well as that the complete final unit is evaluated as a new entity for toxicity [53].

Sound in vivo investigations of biodistribution and toxicology performed prior to translation into the clinic should be the rule. Radiolabeled nanoparticulate administration provided to healthy animals may cause a less severe toxicity profile compared to a severely ill or co-morbid population. Indeed, epidemiological investigations indicate that nanomaterial-related toxicity (with normal pharmaceuticals) occurs mostly in patients with impaired health [53]. Therefore, Table 2 provides a non-exhaustive summary regarding nanoparticle parameters that can contribute to toxicity, including relevant guidelines.

**Figure 4:**
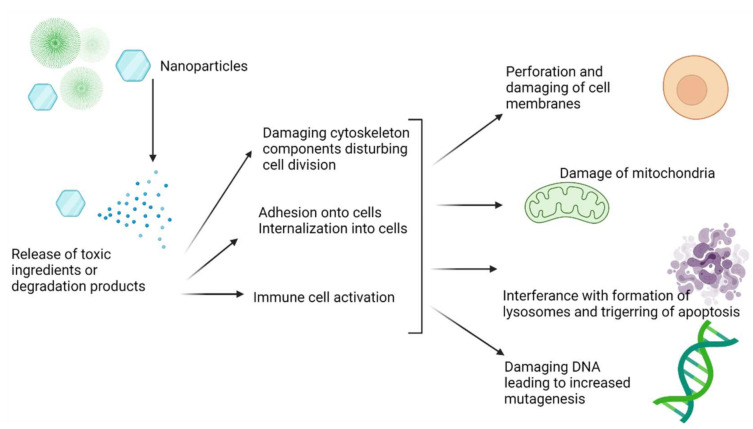
The most common mechanisms causing toxicity associated with nanotechnology [66,67,68] (Figure created with BioRender.com (accessed on 25 April 2021)).

Considering the complexity of the nanoparticle system and the added aspect of radiation, the choice of the correct experimental models is essential to achieve accurate and valuable in vitro and in vivo toxicity data. These should be able to prove beyond doubt that no additional adverse effects may be expected (due to the unique surface properties and construction of these particles). The relevant literature has a well-established database of possible toxicity associated with nanoparticles (Figure 4). A well-known nanotoxicological classification system (proposed by Keck and Muller) puts nanoparticles in four classes of toxicity according to size and biodegradability [64,65]. Additional parameters added are the route of administration (of which intravenous administrations relevant to TAT pose additional risks) and non-biocompatibility surfaces that can activate the immune system [63].

## 6. The Way Forward?

Most of the above-mentioned content is of significant value in TAT as part of the theranostic approach. Theranostics can be seen as a rapidly emerging, elegant concept in nuclear medicine, where the same radiopharmaceutical entity is used for diagnosis (i.e., incorporating a radionuclide with nuclear imaging properties) as well as therapy (i.e., replacing the diagnostic radionuclide with a therapeutic radionuclide). This therapy concept involving α-particle emitters has been well-reviewed in the literature [68,69]. It is also important to note that α-particle emitters have already made a significant impact in theranostics in instances where the condition treated has become refractory to β-emitter therapy [70]. In the search for valuable cancer therapies, there is a wide range of research committed to develop nano-sized theranostics; in particular within nuclear medicine the field of nano-theranostics has not yet gained momentum. According to Drude and co-workers [68], the number of publications in nuclear medical theranostics focusing on nano-theranostics is still small (~3%). This is ascribed to the difficulties in the engineering of biocompatible nano-theranostics with high specificity for malignancies. Nano-theranostics, arguably the most elegant and technologically advanced systems in the field of nanomedicine, can provide real-time information about drug distribution, release, and targeting, which is the embodiment of personalized medicine [71], and more research in this field is necessary. As TAT in nuclear medicine is often hampered by adapting to dosimetry issues [72], combinations with diagnostic modalities can be invaluable. Though yet undeveloped, an exciting emerging advance is the deployment of TAT to address tumor heterogenicity with so-called “smart” or “responsive” theranostics. In this instance, TAT is triggered to release payload in response to: (i) the lower pH occurring in the tumor environment, (ii) cellular conditioning upon reactive oxygen species’ activity, (iii) trigger enzyme activity, or (iv) tumor-specific stimuli [71].

**Figure 5:**
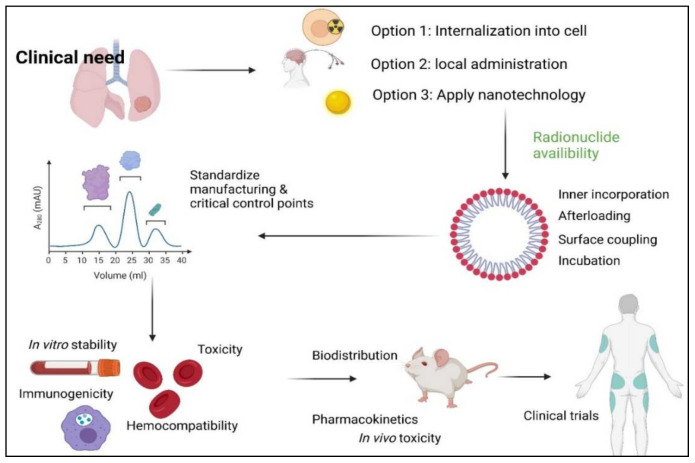
Proposed strategy and milestones for TAT-compliant nanoparticle development [1,57,73,74]. Physicochemical properties: size, shape, chemical composition, physicochemical stability, crystal structure, surface area, surface energy, and surface roughness. Hemocompatibility: RBC lysis, aggregation, and complement activation. Immunogenicity: cytokine modulation and IgG and IgM induction. Pharmacokinetics: chemokine modulation, passage over biological barriers, and whole-body pharmacokinetics.

Proposing new strategies and milestones for an efficient clinical translation of nanoparticle-based TAT is difficult but should ideally start with the objective that the clinical setting is required to address. As pointed out by Coi and Frangioni [66], it should be evaluated if nanomaterial is indeed the only option to solve the clinical problem identified. There are additional complexities introduced by nanoparticles that might pose more problems than other available viable options. As demonstrated in Figure 5, one of three different options may be selected to facilitate strategic decisions on appropriate therapeutic procedures (this is mainly directed to deal with recoil energy). This facilitates the evaluation of: (1) available ligands to assist with quick cell internalization of the radionuclide of choice, (2) loco-regional administration at the tumor, and (3) the radionuclide encapsulation in nanocarriers [1].

It is therefore suggested that cell internalization is investigated as an option where the radiopharmaceutical accumulates inside cancer cells, before nanocarriers are investigated. The second option is loco-regional administration, where the radionuclide is injected directly into the tumors. This technique has been used over the past 5 years with considerable success (e.g., [^213^Bi]Bi-DOTA-Substance P treatment of gliomas) [75]. Finally, if the previous two options are not applicable, the more complex development of nanoparticle-TAT might be an alternative worth exploring. Following that, production of radionuclides is regrettably a limiting factor (which has been reviewed extensively). This can hamper the development of TAT with nanoparticles since it is assumed that more traditional approaches (such as peptide vector delivery) will receive more resources, including scarce radionuclides. Cell internalization and local administration are likely to be chosen first before more advanced and intricate applications such as nano-systems. It is important to note that the use of radium-223, relatively more easily available compared to other alpha emitters, is currently under-utilized because of its poor conjugating chemistry and lack of chelators. Additional applications in TAT for radium-223 may be feasible using nanoparticulate delivery systems. For this reason, nanoparticle-TAT should be focused more on radium-223. Although the chemistry of radium-223 does not make its incorporation into biomolecules relatively easy, its incorporation into nanoparticle systems has been demonstrated successfully. The physical half-life of radium-223 is also especially appropriate for radiolabeling and does not add additional constraints to the manufacturing processes of nanoparticles.

Future developments should include GMP production on a large scale, and standardized quality control assays for characterization should be established. In addition, more specific studies on nanotechnology toxicology are required as it is a significantly more advanced understanding of the interaction of nanoparticles with tissues and cells. Preclinical evaluations are required to characterize the structural stability of nanoparticles within a living organism and adequately determine the accumulation of the nanomedicines in both target and off-target organs. Further barriers for translation into the clinic include a lack of clear regulatory guidelines specifically for nanomedicines, the complexity of patents, and IP, which is a priority for the nuclear medicine community at large. There remains a limited understanding of how nanoparticulate systems behave when administered to patients. Resolution of this issue requires a significant infrastructure and economic backing [61].

## 7. Conclusions

For clinical translation of TAT with nanotechnology to become reality, several hindrances require resolution. These mainly relate to selection of the most suitable nano-platform or -material, incorporating the most suitable radionuclide and subsequently developing an ideal manufacturing procedure with fewer steps, quicker synthesis time, high reproducibility, and lower costs [76]. The most successful current strategy appears to be the utilization of small core nanoparticles that are loaded with the α-emitters manufactured from confining shells with high-Z materials. Regarding improvements in the technology, the most critical parameter will be predicting the adequate thickness of the surrounding shell [55] and to ensure that the persistence in vivo does not elicit any additional toxicity concerns. Overall, current challenges that hamper a nano-pharmaceutical design also hinder developing radiopharmaceuticals involving nanoparticulate systems. Key considerations (in particular order) for the design of radiolabeled nanoparticles are: (A) reducing the complexity in nanoparticulate design and knowledge of possible stressors, (B) need for superior biocompatibility and biodegradability (i.e., pharmaceutical stability), (C) safety of the final dosage form, and (D) a safe (non-toxic) route of administration.

Kozempel and co-workers [52] make a valid point pertaining to the use of TAT, and it is our perception that TAT can be developed further into nanoparticle-TAT and successfully translated to the clinic. It is proposed that the widespread use of TAT in large part also depends on the confidence of the end-users, their understanding of the technology, as well as overcoming the negative historical experiences. It might be prudent to look closely at the multidisciplinary design team at this stage of radiopharmaceutical nanotechnology readiness level and ensure that the right expertise is dealing with bench-to-bedside translation.

## Figures and Tables

**Table 1:** Overview of current preclinical TAT nanotechnology applications.

System	Benefit of Nanotechnology	Labeling Conditions	Findings	Ref.
Astatine-211				
Ultrashort carbon nanotubes	Containment of astatine-211 in a nano-particle that allows binding to targeting molecule	Ultrashort carbon nanotubes were incubated for 10 min with astatine-211 (^211^At^−^) solution at room temperature in both H_2_O and methanol.	Demonstrated entrapment of astatine-211 inside nanotube and in vitro stability.	[14]
Silver containing nanoparticles coated with poly-ethylene oxide	Containment of astatine-211 in a nanoparticle that allows binding to targeting molecule	A solution of sliver nanoparticles was added to astatine-211 as distilled from the target and eluted in methanol, and left to react for 15 min.	Labeling yields in different conditions were found to be between 50% and 97%. No in vitro studies performed.	[15]
Trastuzumab-gold nanoparticles labeled with astatine-211	Containment of astatine-211 in a nanoparticle that allows binding to targeting molecule	Astatine-211 (^211^At^−^) was added to AuNP-S-PEG-trastuzumab bioconjugate and stirred for 1 h at room temperature. This method made use of adsorption.	Demonstrated in vitro stability as well as high affinity and cytotoxicity towards HER-2-overexpressing SKOV-2 cells.	[19]
Gold nanoparticle for astatine-211 delivery	Labeling of biomolecules with astatine-211 by applying gold nanoparticles	Prepared by adsorption of astatine-211 (^211^At^−^) on already produced and analyzed gold nanoparticles (in deionized water). Process took ± 1 h.	Demonstrated in vitro stability in human fluid and cerebrospinal fluid for 24 h. Hight cytotoxic effect on in vitro glioma cells.	[20]
Actinium-225				
Lanthanum phosphate nanoparticles	Enhanced retention of actinium-225 and decay daughters	Actinium-225 was incorporated during production in the form of [^225^Ac]AcCl_3_. Process took <4 h and nanoparticles were made by precipitation.	mAb 201B—targeting mouse lung endothelium	[21]
Gold nanoparticle [^225^Ac]Ac-AU@TADOTAGA	Brachytherapy IV and IT for injectable radiopharmaceutical	Au@TADOTAGA gold particles and [^225^Ac]AcCl_3_ were incubated at 70 °C for 30 min. The nanoparticles are designed with a chelator to incorporate the metal radionuclide.	U87MG tumor-bearing mice—controls inadequate	[22]
Antibody conjugated liposomes	Increase delivery of actinium-225 to target tumor	Active loading of actinium-225 with ionophores. Incubation times differed from 15 to 60 min with DOTA inside the liposome as chelator. Unlabeled actinium-225 was removed with DTPA from the mixture.	Actiniuum-225 retention as high as 81% ± 7% was obtained. No in vitro tests performed.	[23]
Core–shell Gd_0.8_Eu_0.2_VO_4_ NPs doped with actinium-225	Enhanced retention of actinium-225 and decay daughters	The actinium-225 was incorporated from the start of the synthesis and crystallization of nanoparticles. The radionuclide is in cationic chemical form.	Luminescence and magnetic functionalities demonstrated and radionuclide retention.	[24]
InPO_4_ containing polymersomes	Enhanced retention of actinium-225 and decay daughters	Actinium-225 (dry actinium-225 nitrate) was incorporated in an ionophore film (dissolving the actinium-225 in this solution), this was incubated with InPO_4_ nanoparticles for 1 h.	Increased retention of francium-221 and bismuth-213	[25]
Multivesicular liposomes conjugated to HER2/neu antibody	Enhanced retention of actinium-225 and decay daughters	Actinium-225 was entrapped passively in lipids resuspended with PBS containing [^225^Ac]Ac-DOTA. Note that direct labeling of antibodies will cause damage since acintium-225-DOTA labeling happens at higher temperatures. DOTA act as chelator for the metal radionuclide.	HER2/neu antibody targeting ovarian cancer cells (SKOV3-NMP2) deliver higher fractions of generated alpha particles	[26,27]
PEGylated liposomes targeted with antihuman PSMA J591 antibody or A10 PSMA aptamer	Optimizing biodistribution due to the ease of modification of liposomes	Actinium-225 was loaded in preformed DOTA-containing liposomes mediated by ionophores. Efficient loading took place at 1 h and a temperature of 65 °C. DOTA act as chelators for the metal radionuclide.	J591-labeled liposomes demonstrated higher specific binding to all cell lines compared with A10 aptamer-labeled liposomes	[28]
Actinium-225 loaded in polymersomes	Enhanced retention of actinium-225 and decay daughters	Polymersomes with encapsulated DTPA was incubated for 30 min with actinium-225 ([^225^Ac]AcCl_3_).	Displays little loss of daughters if no recoil is present.	[29]
Peptide-based nano-assembly	Enhanced retention of actinium-225 and decay daughters	The peptides where mixed and dried. After drying, peptides were incubated with actinium-225 and DOTA for 2 h in an ammonium acetate buffer.	Branched amphiphilic peptide capsules that self-assemble and retainment of bismuth-213 in vivo.	[30]
Actinium-225-labeled αvβ3-specific liposomes	Targeting alpha therapy to the blood–brain barrier	The prepared liposomes were incubated with actinium-225 ([^225^Ac]AcCl_3_) at 70 °C for 50 min.	Demonstrated in vivo therapeutic efficacy in orthotopic glioblastoma	[31]
Sterically stabilized liposomes coated with folate-F(ab’)_2_	Enhanced retention of actinium and radium and decay daughters	Prepared by loading preformed liposomes with a Ca-ionophore. Incubation with the radionuclide was done for 30 min at 65 °C. Quenching of the reaction was done by adding EDTA and PBS to the mixture.	Radionuclide-loaded liposomes demonstrated serum stability in vitro	[32]
Thorium-227				
Core–shell Gd_0.8_Eu_0.2_VO_4_ NPs doped with thorium-227	Enhanced retention of thorium-227 and decay daughters	The thorium-227 was incorporated from the start of the synthesis and crystallization of nanoparticles.	Luminescence and magnetic functionalities demonstrated and radionuclide retention.	[33]
Radium-223/radium-224				
Combination chemotherapeutic radium-223 liposomes	Combining chemotherapeutic agent (doxorubicin) with radium-223 therapy	A calcium ionophore was used to entrap radium-223 in preformed liposomes. Entrapment took ±1 h. Radium-223 was added in a solution of sucrose and HEPES.	Liposomal radium-223 was stable in vivo and demonstrated potential.	[34]
Functionalized nanoparticles based on BaSO_4_ to allow binding of radium-223 to targeting molecule	Allow binding of radium-224 to targeting molecules through a functionalized liposome	Nanoparticles are obtained after reprecipitation [^224^Ra]Ra(NO_3_)_2_. Precipitation takes 15 min, after which particles are centrifuged for 15 min.	Show stability of >90% regarding radiometal release from BaSO_4_ matrix. Not evaluated in vivo.	[35]
Nanzeolite particles conjugated to Substance P labeled with radium-223	Allow binding of radium-223 to targeting molecule through a functionalized nanoparticle	Nanozeolite particles were sonificated in a radium-223 solution for 15 min, stirred for another 2 h, and centrifuged for 10 min. The ^223^Ra(NO_3_)_2_ was used to produce ^223^Ra^2+^ cations.	Demonstrated being a viable technology retaining radium-223 for up to 6 days in vitro and retaining 90–95% of daughter decay products.	[36]
SPIONs to drive the therapeutic delivery of radium-223 by magnetic field gradients	Allow binding of radium-223 to magnetic particle that can be targeted	Evaporate radium-223 solution (^223^Ra(NO_3_)_2_) to dryness. Expose nanoparticles for 30–60 min to radium-223 for labeling.	Not tested in vivo, it demonstrated good stability in vitro and high radiolabeling yields.	[37]
Lead-212				
Lead-212 internalized into surface-DTPA liposomes	To use the parent radionuclide lead-212 to deliver bismuth-212 to the target area.	A two-step preparation process: label surface-DTPA liposome with indium and then incubate with lead-212, which naturally passes through the liposome and is entrapped by internal DTPA.	A novel method for entrapment of 2–3 lead atoms per liposome with a yield of 75%. No in vitro testing performed.	[38]
Sterically stabilized liposomes	Minimize the escape of daughter bismuth-212 after decay from lead-212.	Ionophore-mediated loading of lead-212 into preformed liposomes (incubated for 30 min).	Retention of lead-212 and bismuth 212 was 95% after incubation for 20 h at 37 °C in serum.	[39]
Sorption and co-crystallization of lead-212 with nanohydroxyapatite	Transporting lead-212 to the targeting tumor.	Co-crystallization of the lead-212 during nanohydroxyapatite production.	Various parameters in production of nanohydroxyapatite with lead-212 was investigated. No in vitro testing was performed.	[40]
Fullerenes containing lead-212	Reduce myelotoxicity resulting from accumulation of lead-212 in the bone marrow.	Incorporation into fullerene by recoil of polonium-218 parent.	Lead-212 fullerene resulted in inhibition of accumulation of lead-212 to the bone in vivo.	[41]

**Table 2:** Parameter-dependent nanoparticle-derived toxicity [53,63,64,65,66] (Figures created with BioRender.com (accessed on 18 April 2021)).

NP Parameter	Status	Current Guidelines
**Size** 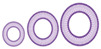	Toxicity influenced by surface-to-volume ratio.	NPs < 50 nm reach tissue faster—more acute toxic effects expected, NPs < 50 nm are used by the RES—however liver and spleen have oxidative stress.
**Shape/Structure** 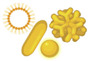	Tissue toxicity is influenced by spherical or rod-like filament or plate-shaped structure	Non-spherical NPs are more exposed to blood flow and exhibit increased toxicity effects regarding hemolysis and/or platelet aggregation.
**Surface** 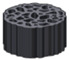	Toxicity caused by different NP surfaces (altered absorption and/or interference with cell adhesion)	Cationic NPs could cause hemolysis and blood clotting, while anionic particles are non-toxic. Zwitterionic and neutral organic surface coatings can reduce penetration of nanoparticles through cellular membranes.
**Concentration** 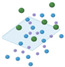	Toxicity caused by NP density (total particles/dose)	The risk for toxic side-effects rises with a higher concentration/density of NP.
**Formulation** 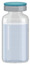	Toxicity can arise from the NP carrier (incompatible organic or inorganic materials).	Composition of non-toxic materials/carriers should be prioritized. Whilst biodegradability can afford issues with stability in vivo, when using non-biodegradable systems, persistence should be monitored to ensure no long-term toxic effects are associated with this. The stable binding of therapeutic radionuclides to nanoparticles (including decay daughters) is essential.

Abbreviations: NP—nanoparticle, RES—reticuloendothelial system.

## Data Availability

No new data were created or analyzed in this study. Data sharing is not applicable to this article.
